# Adjusting the scent ratio: using genetically modified *Vitis vinifera* plants to manipulate European grapevine moth behaviour

**DOI:** 10.1111/pbi.12767

**Published:** 2017-07-18

**Authors:** Umberto Salvagnin, Mickael Malnoy, Gunda Thöming, Marco Tasin, Silvia Carlin, Stefan Martens, Urska Vrhovsek, Sergio Angeli, Gianfranco Anfora

**Affiliations:** ^1^ Faculty of Science and Technology Free University of Bozen‐Bolzano Bolzano Italy; ^2^ Research and Innovation Centre Fondazione Edmund Mach S. Michele all'Adige (TN) Italy; ^3^ Norwegian Institute of Bioeconomy Research, NIBIO Ås Norway; ^4^ Swedish University of Agricultural Sciences Alnarp Sweden; ^5^ Center Agriculture Food Environment (CAFE) University of Trento S. Michele all'Adige (TN) Italy

**Keywords:** *Lobesia botrana*, sesquiterpenes, (*E*)‐β‐caryophyllene, (*E*)‐β‐farnesene, host selection, *Vitis vinifera*

## Abstract

Herbivorous insects use olfactory cues to locate their host plant within a complex olfactory landscape. One such example is the European grapevine moth *Lobesia botrana,* a key pest of the grape in the Palearctic region, which recently expanded both its geographical and host plant range. Previous studies have showed that a synthetic blend of the three terpenoids, (*E*)‐β‐caryophyllene, (*E*)‐β‐farnesene and (*E*)‐4,8‐dimethyl‐1,3,7‐nonatriene (DMNT), was as attractive for the moth as the complete grape odour profile in laboratory conditions. The same studies also showed that the specific ratio of these compounds in the grape bouquet was crucial because a percentage variation in any of the three volatiles resulted in almost complete inhibition of the blend's attractiveness. Here, we report on the creation of stable grapevine transgenic lines, with modified (*E*)‐β‐caryophyllene and (*E*)‐β‐farnesene emission and thus with an altered ratio compared to the original plants. When headspace collections from these plants were tested in wind tunnel behavioural assays, they were less attractive than control extracts. This result was confirmed by testing synthetic blends imitating the ratio found on natural and transformed plants, as well as by testing the plants themselves. With this evidence, we suggest that a strategy based on volatile ratio modification may also interfere with the host‐finding behaviour of *L. botrana* in the field, creating avenues for new pest control methods.

## Introduction

Terpenoids constitute the biggest class of plant metabolites involved both in primary (Croteau *et al*., 2000) and secondary metabolism (Zwenger and Basu, 2008). All terpenes formally derive from the C_5_ isomers isopentenyl diphosphate (IPP) and dimethylallyl diphosphate (DMAPP), whose biosynthesis can take place in plants via two distinct pathways: the mevalonate pathway (MVA) in the cytoplasm and the MEP/DOXP pathway in the chloroplast (Rohmer, 1999). Condensation of C_5_ isomers by prenyltransferase enzymes leads to the C_10_, C_15_, C_20_, C_30_ and C_40_ precursors of mono‐, sesqui‐, di‐, tri‐ and tetraterpenes, respectively. In the last step of the pathway, precursors are converted to terpenes by terpene synthase (TPS) enzymes, which are usually coded by gene families of 20–150 members in each species (Chen *et al*., 2011). In nature, terpenes have many ecological roles, such as direct and indirect defence against pathogens and insects (Hasegawa *et al*., 2010; Heiling *et al*., 2010; Huang *et al*., 2012; Unsicker *et al*., 2009), attraction of pollinators (Dudareva and Pichersky, 2000) and mutualistic fungi (Ditengou *et al*., 2015), as well as being used as signals for plant‐to‐plant communication (Arimura *et al*., 2000). As semiochemicals, their emission is often exploited by phytophagous insects, which use them as kairomones to recognize and locate their host plants (Bruce *et al*., 2005). This is also the case for the European grapevine moth *Lobesia botrana* (Den. & Schiff.) (Lepidoptera, Tortricidae), a polyphagous insect which is considered the main pest of European vineyards and which has the potential to become an invasive species, as recently observed in United States, Argentina and Chile (Ioriatti *et al*., 2011). Worldwide in fruit production, grapes are in first place in terms of crop surface area, with over 3 200 000 ha cultivated and more than 26 Mtons of fruit production in Europe (FAOSTAT, data until 2013, http://faostat.fao.org/). This causes additional concern with respect to *L*. *botrana* management. Control strategies against this pest still primarily involve pesticides, with environmentally friendly techniques such as mating disruption covering less than 4% of European vineyards (Ioriatti *et al*., 2011). With a view to using semiochemicals against insect pests (Birkett and Pickett, 2014), grapevine VOCs involved in host–plant interactions were studied for *L. botrana*, and it was found that a specific blend of the terpenoids (*E)‐*β‐caryophyllene, (*E)‐*β‐farnesene and the homoterpene (*E)*‐4,8‐dimethyl‐1,3,7‐nonatriene (DMNT) was attractive in laboratory and field conditions (Anfora *et al*., 2009), and the attractiveness was shown to be dependent on the kairomone ratio, decreasing significantly when deviating from the ratio found in the grapevine headspace collection (Tasin *et al*., 2006).

In this work, we generated stable grapevine transgenic lines with altered (*E)‐*β‐caryophyllene and (*E)‐*β‐farnesene emission compared to original unmodified plants. In the first case, we overexpressed a grapevine (*E)‐*β‐caryophyllene synthase gene, while in the second case we inserted and express a sweet wormwood gene that codes for an (*E)‐*β‐farnesene synthase. Thus, we modify the ratio between these two kairomones *in vivo*, and tested how it affected *L. botrana* behaviour.

## Results

### Creation of grapevine lines with altered (*E)‐*β‐caryophyllene and (*E)‐*β‐farnesene volatile emission

To modify sesquiterpene emission, both TPS gene overexpression and silencing approaches were considered. In grapevine, there are five genes known to code for (*E)‐*β‐caryophyllene synthase (Martin *et al*., 2010) although *VvGwECar2* (GenBank: HM807374) alone is responsible for most of the volatile production in the green tissues of the plant (Matarese *et al*., 2014). It was therefore chosen as the target for gene silencing using RNAi. However, after plant transformation and regeneration, we could not obtain any significantly silenced line (data not shown), probably because of the difficulty in specifically targeting only one TPS gene in the whole grapevine gene family, in which there is a high degree of sequence similarity among members. By contrast, overexpression of *VvGwECar2* was successful and resulted in the regeneration of many independent lines, as we previously reported (Salvagnin *et al*., 2016).

In order to modify (*E)‐*β‐farnesene emission, we could neither silence nor overexpress any grapevine gene, since apparently there are no known (*E)‐*β‐farnesene synthases in the grapevine genome. The small amount of (*E)‐*β‐farnesene emitted by the plant is indeed the result of the activity of a (*E)‐*α‐bergamotene and a β‐curcumene synthase, that also produce (*E)‐*β‐farnesene as a minority by‐product (Martin *et al*., 2010). Thus, we decided to insert a gene from the sweet wormwood *Artemisia annua* L. which had already been characterized (Picaud *et al*., 2005) as an (*E)‐*β‐farnesene synthase (Aaβ‐FS, GenBank: AY835398.1), and drive its expression with the strong CaMV 35S promoter. All the transformations were carried out as described in Dalla Costa *et al*. (2014), starting from ‘Brachetto Grappolo Lungo’ grapevine variety embryogenic calli (Martinelli *et al*., 2001), and resulted in a dozen‐independent transgenic lines after 1 year. A subset of the lines was extensively propagated and acclimatized in the greenhouse and was used for all the experiments. Overall, we did not notice any difference in plant phenotypes or growth speed in the different lines or as compared to the controls.

**Figure 1:**
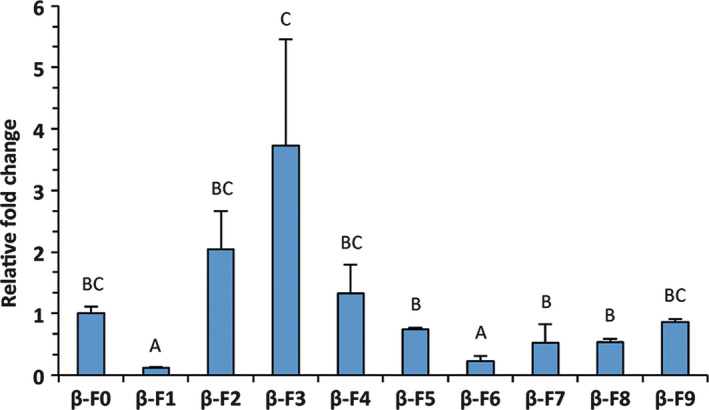
*Aa*β*‐FS* relative expression obtained using RT‐QPCR. Means of reference line β‐F0 were set to 1. At least three biological replicates were used for each line. Letters on bars indicate different groups, according to one‐way ANOVA followed by Fisher's LSD *post‐hoc* test (*P* < 0.05) with standard deviations (SDs) visible for each line.

**Figure 2:**
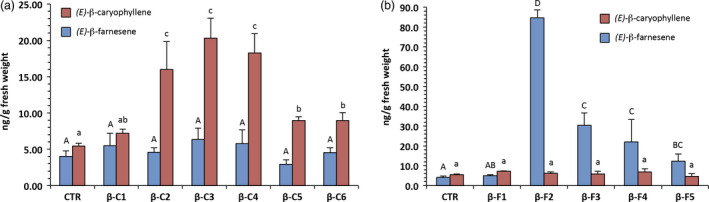
Volatile collection and analysis using SPME‐GC‐MS from young grapevine leaves taken from plants acclimatized in the greenhouse. (a) *(E)‐*β‐caryophyllene overexpressing lines and (b) *(E)‐*β‐farnesene overexpressing lines. Letters on bars indicate different groups according to one‐way ANOVA followed by Fisher's LSD *post‐hoc* test (*P* < 0.05) with the standard error of the mean (SEM) visible for each line.

As frequently observed with random integration of T‐DNA, transgene expression levels varied among lines (one‐way ANOVA, *F* = 6.37; df = 24; *P* < 0.001) when measured with RT‐QPCR (Figure 1), with a 30‐fold difference in transcript level between the weakest and strongest overexpressing lines. To check for changes in sesquiterpene emission, we screened the plants with solid‐phase microextraction and subsequent gas‐chromatography/mass spectrometry (SPME GC‐MS) (Figure 2).

Solid‐phase microextraction was chosen because of its speed and sensitivity, considering that sesquiterpenes usually cover no more than 1% of the total VOCs emitted by the grapevine (Matarese *et al*., 2014). Young leaves of the control plants had a *(E)‐*β‐caryophyllene content in the range of 5 ng/g of fresh weight and *(E)‐*β‐farnesene in the range of 4 ng/g. As previously reported (Salvagnin *et al*., 2016), *(E)‐*β‐caryophyllene overexpressing lines (βC1 to 6) showed an increase of up to 20 ng/g of fresh weight in terms of *(E)‐*β‐caryophyllene content (one‐way ANOVA, *F* = 11.92; df = 20; *P* < 0.001), which was poorly correlated with the gene transcript level. *(E)‐*β‐farnesene overexpressing lines (βF1 to 5) showed a more pronounced increase (one‐way ANOVA, *F* = 18.54; df = 18; *P* < 0.001), with up to 20 times more *(E)‐*β‐farnesene, and a level of 85 ng/g of fresh weight. The correlation between gene expression and metabolites was also higher than the βC lines (Pearsons's r = 0.65). The linearity of SPME fibre response within this concentration range was tested with calibration lines from pure standards, which were also used for quantification. It is worth noting that in βC lines the level of *(E)‐*β‐farnesene was not statistically different from that of control plants (one‐way ANOVA, *F* = 0.66; df = 22; *P* = 0.68), and that in βF lines the level of *(E)‐*β‐caryophyllene was also not statistically different from that of control plants (one‐way ANOVA, *F* = 0.77; df = 21; *P* = 0.59). In other words, a change in the abundance of one volatile did not affect the abundance of the other.

### Plant headspace extraction with closed‐loop stripping analysis

**Figure 3:**
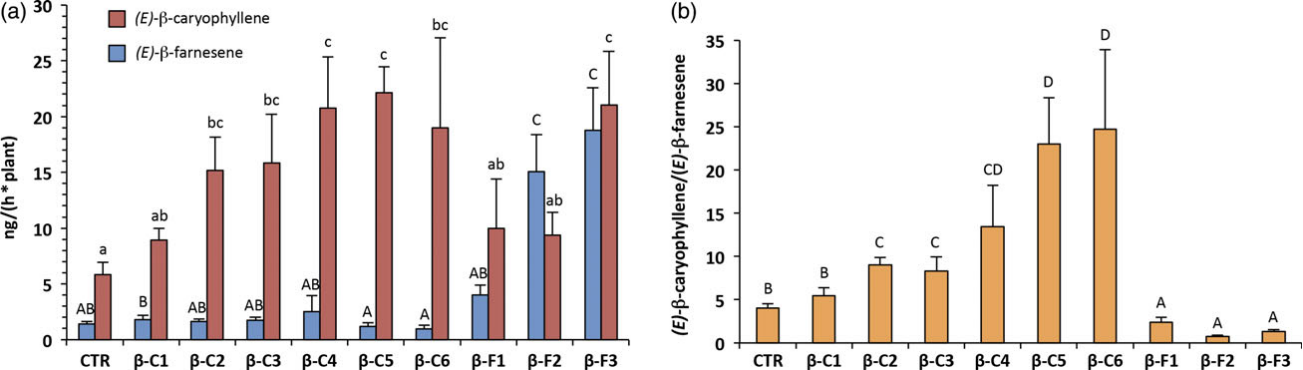
Volatile collection and analysis of the whole above‐ground part of grapevine plants using CLSA‐GC‐MS. (a) Quantification of *(E)‐*β‐caryophyllene and *(E)‐*β‐farnesene emission from the different lines. (b) *(E)‐*β‐caryophyllene/*(E)‐*β‐farnesene mass ratio in the same lines. Letters on bars indicate different groups according to one‐way ANOVA followed by Fisher's LSD *post‐hoc* test (*P* < 0.05) with the standard error of the mean (SEM) visible for each line.

Although solid‐phase micro‐extraction (SPME) allowed us to have fast confirmation of the plant phenotype, it also had the limitation of basing the sampling process only on a few young leaves from an entire plant. Indeed, terpenoid emission is known to vary significantly in the different organs of *V. vinifera* (Matarese *et al*., 2014) and in the field insects are likely to choose their host plant based on VOC emission from the whole body of the plant, rather than from just a few leaves. For this reason, we decided to extract and characterize the plant headspace through closed‐loop stripping analysis (CLSA) (Abraham *et al*., 2014; Boland *et al*., 1984). This method also has the advantage of being non‐destructive, and is carried out at environment temperature, thus not altering plant physiology and more reliably reflecting the emission of VOCs in real ecological conditions. Sampling was done on plants of the same age and of a similar size (30–35 leaves) and was started at the same time every day to exclude the possible effects of the day/night cycle on VOC emission (Chalal *et al*., 2015; Giacomuzzi *et al*., 2016). Quantification of *(E)‐*β‐caryophyllene and *(E)‐*β‐farnesene (Figure 3a) confirmed the pattern observed with SPME quantification although it showed greater variability between biological replicates, and in some lines (βC5, βC6 and βF3) it indicated that SPME sampling had rather underestimated VOC emission. Overall, our initial goal of obtaining grapevine plants with an *(E)‐*β‐caryophyllene/*(E)‐*β‐farnesene emission ratio divergent from the wild type was achieved (one‐way ANOVA, *F* = 24.07; df = 45; *P* < 0.0001), with extracts ranging from 0.22 : 1 to 38 : 1, while the control plants were in the range of 4 : 1 (Figure 3b).

### Wind tunnel behavioural assays

**Figure 4:**
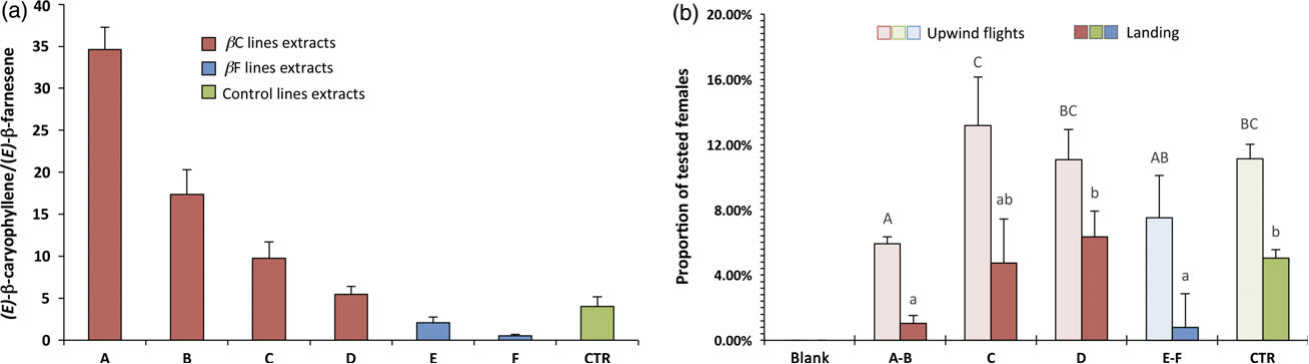
Wind tunnel behavioural assays. (a) Plant extracts used in the wind tunnel sorted by *(E)‐*β‐caryophyllene/*(E)‐*β‐farnesene ratio, with each group separated using Fisher's LSD *post‐hoc* test (*P* < 0.05), except for groups D and CTR, which are not statistically different. The standard deviation (SD) for each category is visible. (b) Attraction (upwind in light and landing in dark) elicited on 3‐day‐old mated *Lobesia botrana* females. Blank: *N* = 60; A–B: *N* = 432; C: *N* = 207; D: *N* = 316; (E)–F: *N* = 207; CTR:* N* = 231. The standard error of the mean (SEM) is visible for each category and letters on bars indicate different groups according to one‐way ANOVA followed by Fisher's LSD *post‐hoc* test (*P* < 0.05).

All the extracts were sorted and grouped into seven categories (Figure 4a) according to their *(E)‐*β‐caryophyllene/*(E)‐*β‐farnesene ratio: six categories (A to F) from transformed plants with increasingly different ratios (from 35 : 1 to 0.5 : 1) and one category (CTR) with extracts only from control plants (ratio = 4 : 1 on average).

Categories A, B and C, with a higher ratio as compared with the controls, were obtained from βC lines, while categories E and F, with a lower ratio compared to the controls, were obtained from βF lines. Category D extracts were obtained from βC lines whose kairomone emissions were not statistically different from the CTR category, and were thus used as a further control. It is worth noting that the ratio found on control plants of our variety (4 : 1) was different from the ratio (10 : 1) found in previous studies (Tasin *et al*., 2006), where ‘Casana’ plants were used, highlighting the varietal variation.

Extracts from all the groups were tested for attraction in wind tunnel assays (Figure 4b) using adult *L. botrana* mated females 3 days after eclosion, reared on a semi‐artificial diet. Responding insects took flight from the tubes on the downwind side of the wind tunnel and locked into the odour plume, starting to fly upwind. The maximum linear flying distance that an insect could cover before hitting the protective cage in front of the sprayer was 180 cm: any flight between 50 cm and 170 cm from the odour source was defined as upwind flight, while arrival at any point within 10 cm of the sprayer was defined as landing (Figure S1). The insect response rate was low, but in line with previous studies performed on this species when reared in semi‐artificial conditions (Tasin *et al*., 2005), and it proved to be consistent over time within the same groups of extracts and throughout all the batches of insects tested. Extracts from control plants elicited on average 11% of upwind flights and 5% of landings. Group D elicited the same attraction as compared to the CTR group, both in terms of upwind flights and landings, as could be expected, given that the VOC ratio was not statistically different. Group C contained extracts from plants which weakly overexpress *(E)‐*β‐caryophyllene, with an average emission ratio close to 10 : 1. This ratio is different from that of the ‘Brachetto Grappolo Lungo’ variety wild type, but not from that of the ‘Casana’ variety used in previous studies, and indeed all the extracts tested from group C elicited attraction very similar to group D and the CTR group (Figure 4b). Groups A, B, E and F were the most divergent from the CTR group, with all ratios higher than 17 : 1 or lower than 2 : 1. These groups all showed a modest decrease in the number of upwind flights (one‐way ANOVA, *F* = 4.49; df = 15; *P* = 0.021) and a major decrease in the number of landings (one‐way ANOVA, *F* = 4.02; df = 20; *P* = 0.019), with less than 1% of the total insects tested. Moreover, specifically with respect to the groups of extracts A and F, we observed some differences in the way insects flew towards the sprayer: when leaving the tubes downwind they seemed to maintain casting behaviour for a longer time, and they frequently lost the odour plume before giving up and resting on the walls of the tunnel.

**Figure 5:**
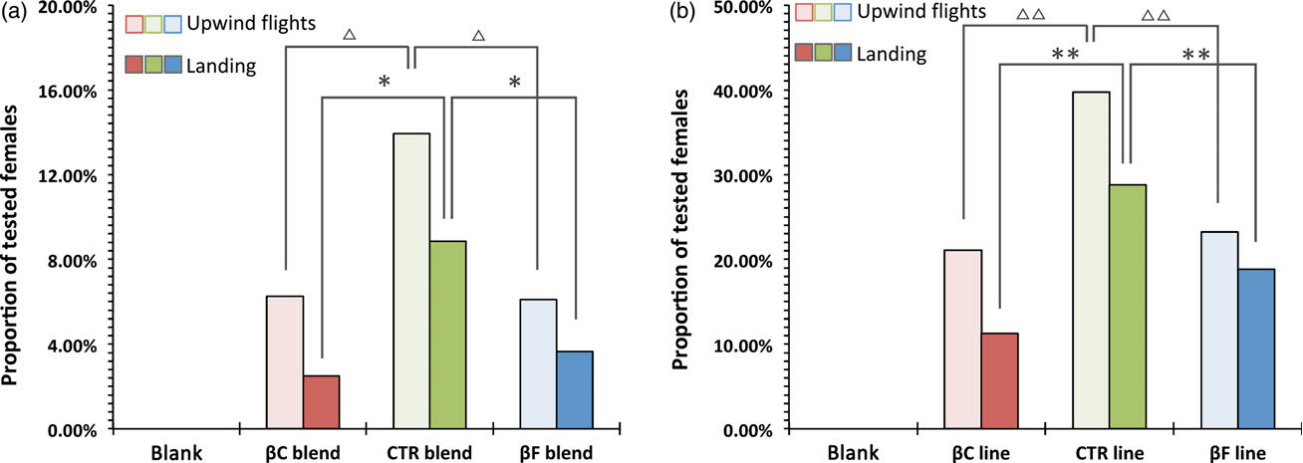
Wind tunnel behavioural assays. (a) Attraction (upwind in light and landing in dark) elicited on 3‐day‐old mated *Lobesia botrana* females from synthetic three‐component blends that mimic, respectively, the βC, CTR and βF profile. Blank: *N* = 60; βC blend: *N* = 80; CTR blend: *N* = 79; βF blend: *N* = 82. Symbols indicate statistical significance: ▵∶ χ^2^ = 7.65;df = 2; *P* < 0.05 and * ∶χ^2^ = 6.76;*df* = 2; *P* < 0.05. (b) Attraction (upwind in light and landing in dark) elicited on 3‐day‐old mated *Lobesia botrana* females from real plants. Blank: *N* = 39; βC line: *N* = 71; CTR blend: *N* = 73; βF line: *N* = 69. Symbols indicate statistical significance: ▵▵∶ χ^2^ = 13.92;*df* = 2; *P* < 0.001 and **χ^2^ = 18.14;df = 2; *P* < 0.001.

To confirm that the different behaviour of the moths was due to the changing ratio between *(E)‐*β‐caryophyllene and *(E)‐*β‐farnesene, we created three synthetic blends based on the original three‐component attractive lure (Tasin *et al*., 2006). The control (CTR) blend mimicked the ratio of control plants, which for our variety was 4 : 3.3 : 1 for *(E)‐*β‐caryophyllene, DMNT and *(E)‐*β‐farnesene, respectively, while the βC blend had a ratio of 25 : 3.3 : 1 (similar to βC5 plants) and the βF blend had a ratio of 0.3 : 3.3 : 1, similar to βF2 plants. The blends were sprayed into the wind tunnel at a rate of 35 ng/h of the main compound, and female attraction was scored in the same way as was measured with plant extracts (Figure 5a).

Insect response percentages were very similar to those found with headspace collections, and both the βC and βF blends were less attractive than the CTR blend (upwind: χ^2^ = 7.65; df = 2; *P* < 0.05; landing: χ^2^ = 6.76; df = 2; *P* < 0.05).

Finally, to confirm the difference in behaviour, we tested two representative plant lines, βC5 and βF2, in the wind tunnel, and compared their attractiveness to the CTR line (Figure 5b). All the plants were of the same age, grown in the same conditions and had a similar number of leaves (about 80). Insect response percentages here were much higher than in previous experiments, probably because the plants had a higher emitting surface area as compared to the sprayer, and their odour profile was not affected by any sampling processes. In any case, the trend observed remained the same, with lower attractiveness for plants with an altered *(E)‐*β‐caryophyllene/*(E)‐*β‐farnesene ratio as compared to the control, both in terms of upwind flights (χ^2^ = 13.92; df = 2; *P* < 0.001) and landing (χ^2^ = 18.14; df = 2; *P* < 0.001).

## Discussion

Herbivorous insects use olfactory cues to locate their host plants in complex environments (Bruce *et al*., 2005). This discrimination can be achieved either by the perception of species‐specific VOCs (Blight *et al*., 1995; Nottingham *et al*., 1991) or more frequently by the presence of a blend of common VOCs emitted in a specific ratio (Thöming and Knudsen, 2014). Studies using synthetic blends in a wind tunnel showed that ratio modification of the components led to a decrease in attractiveness (Bruce *et al*., 2005; Cha *et al*., 2011), suggesting that VOC manipulation could be exploited to establish novel pest control methods (Turlings and Ton, 2006; Unsicker *et al*., 2009). Considering the costs of many VOCs due to difficulties in their synthesis or isolation from natural sources, and considering especially that they often have a short half‐life in nature because of the tendency to form isomers and/or become oxidized (Blande *et al*., 2014), a genetic engineering approach seems most suitable for studying the effect of kairomone manipulation on insect behaviour (Birkett and Pickett, 2014). In plants of agricultural interest, only a few cases of genetic engineering with insect semiochemical biosynthesis have been reported, interestingly regarding the same VOCs we targeted in our study. In maize, it was shown that the expression of a *(E)‐*β‐caryophyllene synthase resulted in high *(E)‐*β‐caryophyllene‐emitting plants that could attract insect‐killing nematodes (Degenhardt *et al*., 2009), suffering less root damage in the field from *Diabrotica virgifera virgifera* as compared to control lines. More recently, the expression of a peppermint *(E)‐*β‐farnesene synthase in wheat led to the creation of high *(E)‐*β‐farnesene‐emitting plants (Bruce *et al*., 2015) with the view to repel aphids, for which *(E)‐*β‐farnesene is an alarm pheromone. In this case, plants were repellent under laboratory conditions for at least three species of cereal aphids although aphid infestations were not reduced during field trials.

In this work, we report for the first time on the transformation of a woody plant species of agricultural interest with a modified kairomonal biosynthetic pathway. In particular, we produced *V. vinifera* plants keeping all the kairomones used by *L. botrana* for host location, but with several degrees of ratio modification between two key components, namely *(E)‐*β‐caryophyllene and *(E)‐*β‐farnesene. Biosynthesis of the third key component DMNT was not a goal of our genetic modification, and its emission remained unaltered in the different lines (one‐way ANOVA, *F* = 1.35; df = 47; *P* = 0.24). The data presented show a certain degree of plasticity in terms of insect choice, considering that mild but significant volatile ratio variations were not sufficient to lose *L. botrana* attraction. The most probable reason for this plasticity is the natural variation in VOC emission in different plants, even when the genetic background remains the same (clones) as well as the environmental conditions. Different genetic backgrounds, as would occur within a natural population and/or in the case of different varieties, is likely to enhance ratio differences. Indeed, as mentioned previously, prior studies using another variety (Tasin *et al*., 2006) found a typical average ratio of 10 : 1, which is the same as for ‘Brachetto Grappolo Lungo’ transformed βC plants falling into category C, whose ratio was on average at least double that of wild types for this variety. However, when the kairomone ratio is very distant from the typical values found in the before‐mentioned varieties (more than three times higher or less than half, as in this case) we measured a clear decrease in attractiveness and an increased difficulty in recognizing the host plant, despite the presence of all the other VOCs in the headspace. Overall, we conclude that modification of the kairomone ratio within the grape bouquet is sufficient to interfere with the host‐finding behaviour of *L*. *botrana*, and that our findings could form the basis for development of new environmentally friendly approaches for pest control. In particular, in *V. vinifera*, apart from keeping the genetic background of elite varieties unaltered, the use of genetically engineered plants could also have the advantage of modifying the behaviour of other grapevine phytophagous insects (e.g. *Eupoecilia ambiguella*,* Scaphoideus titanus*), which may use the same VOCs as part of their kairomonal blend although this remains to be investigated. For the same reason, care should be taken not to underestimate possible negative effects on beneficial insects, which help to naturally contain pest populations in vineyards. We are also aware that another critique issue could be represented by adaptation: considering that learning behaviours were reported for Lepidoptera species (Bruce and Pickett, 2011; Hartlieb *et al*., 1999; Riffell *et al*., 2008), it is probable that *L*. *botrana* could learn to recognize the shift in kairomones ratio, nullifying its effect. For this reason, the engineered grapevine genotypes should be better exploited in integrated pest management (IPM) programmes, where the use of multiple strategies against *L. botrana* could delay the arise of the phenomenon.

## Experimental procedures

### Cloning of the *(E)‐*β‐farnesene synthase into plant overexpression vectors

A 1‐month‐old *Artemisia annua* plant was obtained from seeds (www.worldseedsupply.com) germinated in the greenhouse. Mature leaves were used to extract total RNA (Spectrum^TM^ Plant Total RNA kit, Sigma‐Aldrich Co., St. Luis, MO, USA) and 1.0 μg was retro‐transcribed (SuperScript^®^ III Reverse Transcriptase, Invitrogen, Carlsbad, CA, USA) with the gene‐specific primer ‘aaBfs rev’ (5′‐TTAGACAACCATAGGGTGAACG‐3′). cDNA was used as a template to amplify the whole coding sequence of the *(E)‐*β‐farnesene synthase (Aaβ‐FS, GenBank: AY835398.1) with the forward primer ‘attB‐β‐FS for’ (5′‐GGGGACAAGTTTGTACAAAAAAGCAGGCTTAACAATGTCGACTCTTCCTATTTCTAG‐3′) and the reverse primer ‘attB‐β‐FS rev’ (5′‐GGGGACCACTT TGTACAAGAAAGCTGGGTTTAGACAACCATAGGGTGAACG‐3′). PCR product was cloned directly into pDONR221 via BP‐clonase reaction (Invitrogen), and transformed into *E. coli* DH5α chemically competent cells. Plasmid minipreps from single colony 5 mL cultures were sequenced to check for their quality using the M13(‐20) for (5′‐GTAAAACGACGGCCAG‐3′) and M13 rev (5′‐CAGGAAA CAGCTATGAC‐3′) primer pair. The coding sequenced was then moved into the gateway destination vectors pK7WG2D (Karimi *et al*., 2007) via LR reaction (Invitrogen). 1 μL of the reaction was used to transform chemically competent *E.coli* TOP10 cells (Invitrogen), then plasmid minipreps from single colony 5 mL cultures were sequenced to check for their quality using my35Sprom for the (5′‐CCACTATCCTTCGCAAGACCC‐3′) and my35Sterm rev (5′‐GAAGTATTTTACAAATACAAATACATACTAAGG‐3′) primer pair.

### Grapevine Transformation

The pK7WG2D vector containing the Aaβ‐FS coding sequence was used to transform embryogenic *calli* of *V. Vinifera* (‘Brachetto Grappolo Lungo’ variety) via *Agrobacterium tumefaciens,* as described in Dalla Costa *et al*. (2014). Transgenic plants were propagated and maintained *in vitro* until acclimatization in the greenhouse.

### RNA extraction and RT‐qPCR

RNA was extracted with the Spectrum™ Plant Total RNA kit (Sigma), from young grapevine leaves (second and third internodes below the apex) that had previously been acclimatized in the greenhouse following the manufacturer's guidelines. RNA quality and quantity was checked on a spectrophotometer and 1% agarose electrophoresis before cDNA was retro‐transcribed using SuperScript^®^ III Reverse Transcriptase (Invitrogen) with Random Primers.

The primer pair ‘Aaβ‐FS RT for’ (5′‐TGAGGGTGGAAGATGAAACAATA‐3′) and ‘Aaβ‐FS RT rev’ (5′‐CTTAGGGAAGAGTCACAAGAAGG‐3′) had an efficiency of 94.3% and was used in RT‐qPCR to determine transgene expression.

qPCR was performed on an CFX96 thermocycler (Bio‐Rad, Hercules, CA, USA), using GAPDH and Actin (Reid *et al*., 2006) as reference genes. Real‐time PCR was carried out with the following cycle: 95 °C 10’; 40 x (95 °C 30”, 60 °C 30”); The manufacturer's software (CFX Manager, Bio‐Rad) was used to calculate primer pair efficiency (E) and the threshold cycle (Ct) mean and standard deviation for each sample. Analysis of relative quantification was performed using the workflow reported in Hellemans *et al*. (2007). For each primer pair used, Cts from reference lines were used as references to calculate ΔCt. The geometric mean between the E^▵Ct^ value from Actin and GAPDH was used to calculate a normalization factor (NF), which was finally used to calculate the fold difference according to the formula: $\text{Fold Difference}=E^{▵C_{t}/\text{NF}}$.

### Volatile analysis with SPME‐GC‐MS

Sesquiterpene collection was performed with a method adapted from Matarese *et al*. (2014). A CTC CombiPAL autosampler (CTC Analytics, Zwingen, Switzerland) with a single magnetic mixer (SMM Chromtech, Bad Camberg, Germany) was used to extract the volatiles from the sample vial headspace. A Trace GC Ultra‐gas chromatograph coupled to a Quantum XLS mass spectrometer (Thermo Scientific, Electron Corporation, Waltham, MA) was used: compounds were separated using a VF‐Wax ^®^ (100% polyethylene glycol; 30 m × 0.25 mm × 0.25 μm, Agilent J&W Scientific Inc., Folsom, CA). The GC oven parameters were as follows: initial temperature was 40 °C, maintained for 4 min, followed by an increase to 60 °C at a rate of 2 °C/min, the oven was then maintained at 60 °C for 1 min, then increased at a rate of 5 °C/min up to 190 °C for 1 min and at a rate of 10 °C/min up to 230 °C, maintained for 4 min; splitless time of 5 min and GC inlet temperature of 250 °C. Helium was used as carrier gas in constant flow mode at 1.2 mL/min. The total cycle time was 50 min. The MS detector was operated in scan mode (mass range: 40–450 m/z) with a 0.2 s scan time and the transfer line to the MS system was maintained at 250 °C.

Solid‐phase microextraction extraction was carried out with a slight modification as compared to that described in Matarese *et al*. (2014). Half a gram of leaf powder was added to 0.3 g of NaCl and 3 mL of freshly prepared citrate‐phosphate buffer (0.1 m Na_2_HPO_4_, 50 mm citric acid, pH 5.0) and put into 20 mL glass headspace vials. Each sample was added with 106.5 ng of 2‐octanol as internal standard. Samples were kept at 60 °C for 20 min and then extracted for 35 min at 60 °C. The headspace was sampled using 2‐cm DVB/CAR/PDMS 50/30 μm fibre (Supelco, Bellefonte, PA). The volatile and semi‐volatile compounds were desorbed in the GC inlet at 250 °C for 4 min in splitless mode and the fibre was reconditioned for 4 min at 270 °C.

### Headspace collection and analysis through *CLSA*

Plant headspace was collected using the closed‐loop stripping analysis (CLSA) method (Abraham *et al*., 2014; Boland *et al*., 1984). The above‐ground part of the plant (approximately 30 leaves) was put into a closed plastic cooking bag (25 × 38 cm, Cuki, Italy) where an air pump (DC 12/16 FK, Aersistem, Milano) created a flow of activated‐charcoal‐cleaned air at 0.8 L/min. The air flow passed through an adsorbent activated charcoal filter (CLSA‐Filter, LowResistance 1.8 mg, Brechbühler AG, Switzerland) for 3.5 h, before trapped compounds were eluted with 160 μL of dichloromethane. Before being used again, each filter was cleaned with 6 mL of dichloromethane. At least three acclimatized plants were used as biological replicates for each line tested.

An Agilent 6890N gas chromatograph with autosampler and 5973 mass selective detector was used for separation of liquid injections. Compounds were separated using a J&W Scientific DB‐Wax column (100% PEG; 30 m × 0.25 mm × 0.25 μm, Agilent) and a 2.5 m methyl‐deactivated pre‐column (Varian Inc., Lake Forest, CA) with the same internal diameter that was connected to the analytical column via a press‐fit connector (BGB Analytik AG, Boeekten, Switzerland). The GC oven parameters were as follows: initial temperature was 40 °C, maintained for 2 min, then increased at a rate of 6.9 °C/min up to 160 °C, and then 21.5 °C/min to 250 °C for 3.60 min; injection volume was 1 μL in splitless mode with a GC inlet temperature of 250 °C. Helium was used as carrier gas in constant flow mode at 1.6 mL/min. The total cycle time was 27.18 min. The MS detector was operated in scan mode (mass range: 40–550 m/z) and the transfer line to the MS system was maintained at 250 °C.

### Data processing

For SPME‐GC‐MS data, processing was carried out with XCALIBUR™ 2.2 software provided by ThermoFisher Scientific (Waltham, MA, USA), while for CLSA‐GC‐MS data, processing was carried out with MSD ChemStation E.02.02.1431 (Agilent, Santa Clara, CA, USA). Identification of the volatile compounds was made by injecting pure reference standards, and quantifications were performed after calibration lines had been prepared with the same standards.

### Insects

The *L. botrana* moths used in this research were reared on a semi‐artificial diet in a growth chamber at 23.5 °C and with 65% relative humidity. The photoperiod was 1 : 16 : 1 : 6 h, respectively, for dawn : day : sunset : night light conditions. Larvae were kept in plastic boxes (25 × 18 × 5 cm), where they could feed without limits until they pupated. All the pupae were transferred into meshed plastic boxes (30 × 30 × 30 cm), where the adults emerged. Adults were transferred into mating chambers (12 cm diameter, 22 cm long) and provided with 10% sucrose solution to obtain eggs every day, and 3‐day‐old mated females were used each time for all experiments. The insects never experienced any contact with plants or volatiles from the extracts prior to the experiments, and each female was only used once.

### Wind Tunnel

The wind tunnel used with plant headspace extracts and synthetic blends had a flight section of 67 × 88 × 200 cm and was kept at 23 °C with 55%–65% relative humidity. Air flow through the tunnel was 25 cm/s, and inside the tunnel the light intensity was approximately 10 lux. An ultrasonic sprayer equipped with a conical nozzle (Sono‐Tek corporation, Milton, NY) and connected to a model 102 syringe pump (CMA Microdialysis AB, Solna, Sweden) was used to introduce the volatiles into the tunnel. The tip of the nozzle was installed through a metal grid into the centre of the upwind end of the flight section, 30 cm above the floor, and the tip was covered with a glass cylinder (diam. 10 cm, length 8 cm) and a metal mesh.

Each closed‐loop stripping headspace sample was concentrated under the fume hood to remove most of the dichloromethane, and diluted with pure ethanol to a volume of 1.800 mL, corresponding to 3 h headspace collection time when released at a rate of 10 μL/min in the wind tunnel. Groups of five mated females were placed in glass tubes (12.5 × 2.6 cm) capped with a nylon mesh and transferred into the wind tunnel room at least 3 h before the start of the experiment. The wind tunnel tests started 0.5 h before the start of the scotophase: three tubes with moths were placed in the centre of the downwind end of the tunnel, 30 cm above the floor and 160 cm from the sprayer. Each batch of females was allowed to respond for 20 min, and no more than 3 batches per day were used. The nozzle of the sprayer was cleaned with pure ethanol for 10 min before and after use, as well as between different extracts when sprayed the same day. To prevent any odour contamination, the glass cylinders, glass tubes and metal meshes were heated to 300 °C for 8 h before use. The flight behaviour of the moths was observed during the experiment duration, and the numbers of landings as well as upwind flights were recorded. For each of the tested extracts, between 70 and 90 females were used, on at least two different days, and the order of the stimuli was randomized. The percentage of females responding was transformed to log_10_ values and submitted to one‐way analysis of variance (ANOVA), followed by Fisher's LSD test, with a significance level of 0.05 between groups for multiple comparisons.

The attraction to grapevine plants was assessed in a wind tunnel close to the containment greenhouse, which had a flight section of 63 × 90 × 200 cm and was kept in a room with the same conditions as described above. The plant was put behind a metal grid in the upwind end of the flight section, with the pot wrapped in aluminium foil. The preparation of the experiment was the same as described for the sprayed extracts. Each batch of females was allowed to respond for 10 min, and no more than four batches per day were used. The flight behaviour of the moths was observed during that time, and the numbers of landings as well as upwind flights were recorded. From 70 to 90 females were used for each of the tested plants, on at least three different days, and their order was randomized. The percentage of females responding in the different groups was tested for statistical significance using a chi‐squared test.

## Supporting information


**Figure S1** Sketch of the wind tunnel used in the behavioural experiments. Visual stimuli for insect orientation during flight were placed as paper disks on the floor of the tunnel and paper sheets on the roof.
