# Supplementary material for: Adjusting the scent ratio: using genetically modified Vitis vinifera plants to manipulate European grapevine moth behaviour
Source: Plant Biotechnol J. 2017 Jul 18;16(1):264–71. doi: 10.1111/pbi.12767 (PMC5785346; doi:10.1111/pbi.12767)

**Fig. S1** Sketch of the wind tunnel used in the behavioural experiments. Visual stimuli for insect orientation during flight were placed as paper disks on the floor of the tunnel and paper sheets on the roof.


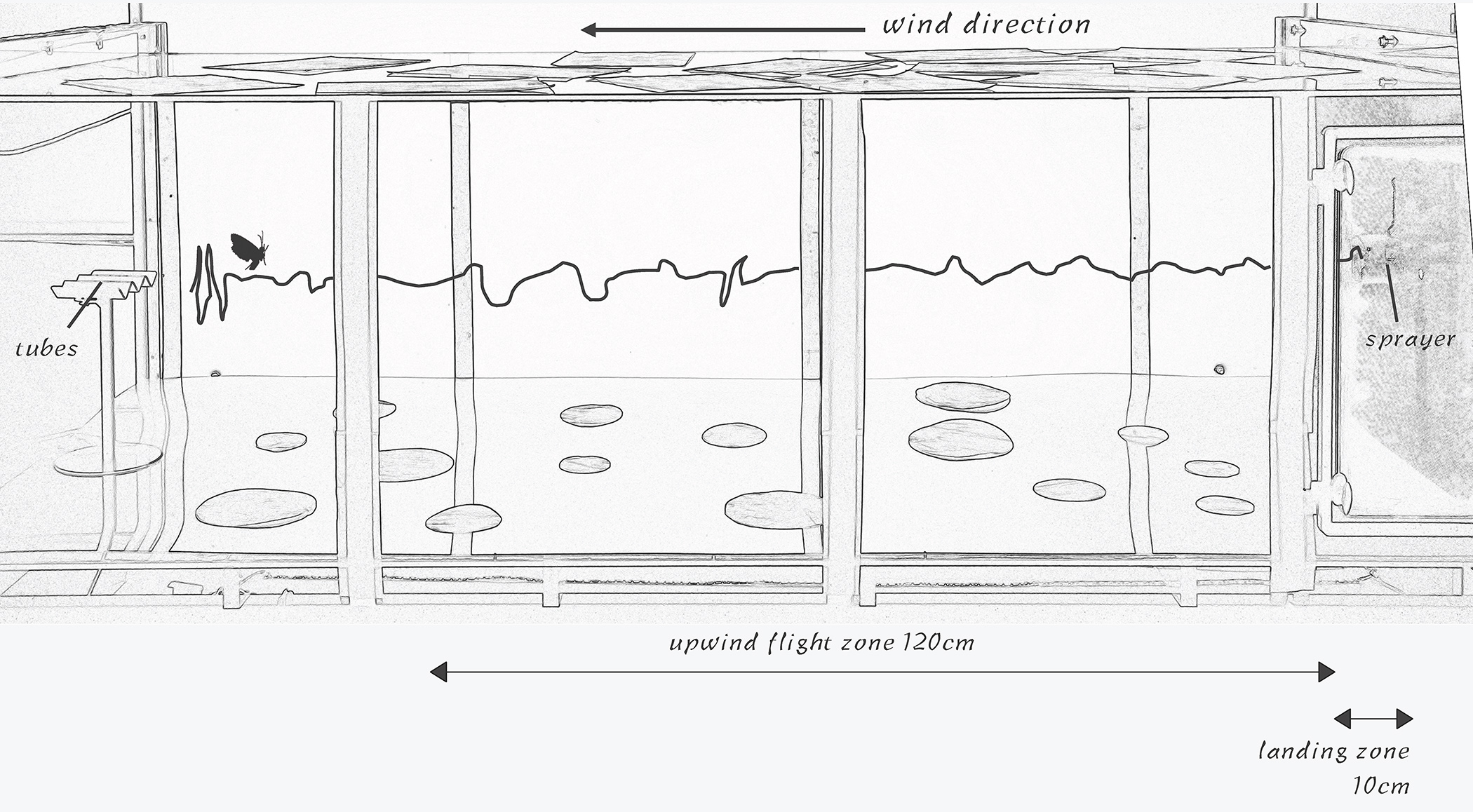

Supplement: Supplementary file 1 — Figure S1 Sketch of the wind tunnel used in the behavioural experiments. Visual stimuli for insect orientation during flight were placed as paper disks on the floor of the tunnel and paper sheets on the roof. [file PBI-16-264-s001.doc]
